# *Mycobacterium tuberculosis* Phosphate Uptake System Component PstA2 Is Not Required for Gene Regulation or Virulence

**DOI:** 10.1371/journal.pone.0161467

**Published:** 2016-08-24

**Authors:** Anna D. Tischler, Rachel L. Leistikow, Pavithra Ramakrishnan, Martin I. Voskuil, John D. McKinney

**Affiliations:** 1 Department of Microbiology and Immunology, University of Minnesota, Minneapolis, Minnesota, United States of America; 2 School of Life Sciences, Swiss Federal Institute of Technology (EPFL), Lausanne, Switzerland; 3 Department of Immunology and Microbiology, University of Colorado Denver School of Medicine, Aurora, Colorado, United States of America; Infectious Disease Research Institute, UNITED STATES

## Abstract

The *Mycobacterium tuberculosis* genome encodes two complete high-affinity Pst phosphate-specific transporters. We previously demonstrated that a membrane-spanning component of one Pst system, PstA1, was essential both for *M*. *tuberculosis* virulence and for regulation of gene expression in response to external phosphate availability. To determine if the alternative Pst system is similarly required for virulence or gene regulation, we constructed a deletion of *pstA2*. Transcriptome analysis revealed that PstA2 is not required for regulation of gene expression in phosphate-replete growth conditions. PstA2 was also dispensable for replication and virulence of *M*. *tuberculosis* in a mouse aerosol infection model. However, a Δ*pstA1*Δ*pstA2* double mutant was attenuated in mice lacking the cytokine interferon-gamma, suggesting that *M*. *tuberculosis* requires high-affinity phosphate transport to survive phosphate limitation encountered in the host. Surprisingly, Δ*pstA2* bacteria were more resistant to acid stress *in vitro*. This phenotype is intrinsic to the alternative Pst transporter since a Δ*pstS1* mutant exhibited similar acid resistance. Our data indicate that the two *M*. *tuberculosis* Pst transporters have distinct physiological functions, with the PstA1 transporter being specifically involved in phosphate sensing and gene regulation while the PstA2 transporter influences survival in acidic conditions.

## Introduction

Phosphorous is an essential element that is required for synthesis of nucleotides, DNA, RNA, phospholipids, and high-energy metabolic intermediates such as acetyl phosphate. Organisms typically acquire phosphorous from the environment as either organic or inorganic phosphate (P_i_) using specific uptake systems. Bacteria use two types of P_i_ acquisition systems that differ in their uptake velocity and substrate affinity. Pit (phosphate inorganic transport) is a low-affinity, high-velocity system that transports metal phosphates (*e*.*g*. Zn^2+^ in complex with P_i_) [1]. Because of its relatively low affinity (*K*_*m*_ = 38.2 μM), the Pit transporter is only functional when P_i_ is in excess [2]. In contrast, the Pst (phosphate-specific transport) uptake system is a high-affinity, low-velocity transporter of free P_i_ [2]. The Pst system can scavenge P_i_ and transport it against steep concentration gradients because the system includes a substrate-binding protein (PstS) that has high affinity for P_i_ and an ATPase (PstB) that provides the energy to drive uptake [2]. Two membrane-spanning components, PstA and PstC, complete the system. The Pst system is functional at P_i_ concentrations as low as 0.4 μM. Therefore, the Pst system is usually required for bacterial survival during conditions of P_i_ limitation.

Bacteria regulate expression of genes that are involved in P_i_ uptake and metabolism in response to the external P_i_ concentration. In the well-characterized *Escherichia coli* model, the transcriptional response to P_i_-limitation is mediated by a two-component signal transduction system PhoR-PhoB. The Pst P_i_ uptake system is essential for inhibiting activity of PhoR-PhoB when the external P_i_ concentration is high [1]. Mutant strains lacking any single component of the Pst system exhibit constitutive activation of the PhoB response regulator and constitutive expression of the Pho regulon [2]. When the external P_i_ concentration is relatively low (≤ 0.4 μM), inhibition of PhoR-PhoB by the Pst system is relieved and the Pho regulon is expressed [1].

We recently demonstrated that the *Mycobacterium tuberculosis* Pst system component PstA1, a membrane-spanning domain of the Pst system, is required for virulence in a murine aerosol infection model [3]. A Δ*pstA1* mutant is sensitive to host immune responses that are dependent on the macrophage-activating cytokine interferon-gamma (IFN-γ). Attenuation of Δ*pstA1* mutant bacteria is partially attributable to a regulatory function of the *M*. *tuberculosis* Pst system. Δ*pstA1* bacteria exhibit aberrant gene expression during growth in medium with high P_i_ concentration; this aberrant transcription is dependent on RegX3, a DNA binding response regulator of the SenX3-RegX3 two-component signal transduction system [3]. RegX3 is required for appropriate regulation of these same genes (activation or repression) during P_i_ starvation. Thus, the *M*. *tuberculosis* Pst system that includes PstA1 functions similarly to the *E*. *coli* Pst system; it inhibits P_i_-starvation responsive SenX3-RegX3 signal transduction when P_i_ is abundant.

*M*. *tuberculosis* is unusual since its genome encodes two complete Pst transporters plus one additional PstS substrate-binding protein [4]. Because PstA1 is required for regulation of gene expression in response to P_i_ availability and for virulence, we wondered if the alternative *M*. *tuberculosis* Pst system might have similar or partially redundant functions. To address these questions, we deleted *pstA2*, which encodes a Pst system membrane-spanning protein paralogous to PstA1, both in a wild-type background and in combination with the *pstA1* deletion. We find that PstA2 does not influence gene expression during growth in P_i_-replete medium. In addition, PstA2 is not required for replication in the lungs of aerosol-infected mice or virulence of *M*. *tuberculosis*. Our results suggest that PstA1 is uniquely required for regulation of gene expression in response to P_i_ and virulence of *M*. *tuberculosis*. The alternative Pst system does, however, play a role in *M*. *tuberculosis* physiology, since we demonstrate that Δ*pstA2* bacteria are more resistant to acidic pH. Our data indicate that components of the two *M*. *tuberculosis* Pst systems are not interchangeable, and suggest that each Pst system has a non-redundant function in *M*. *tuberculosis* physiology.

## Results

### PstA2 Is Not Required for Regulation of Gene Expression

**Fig 1:**
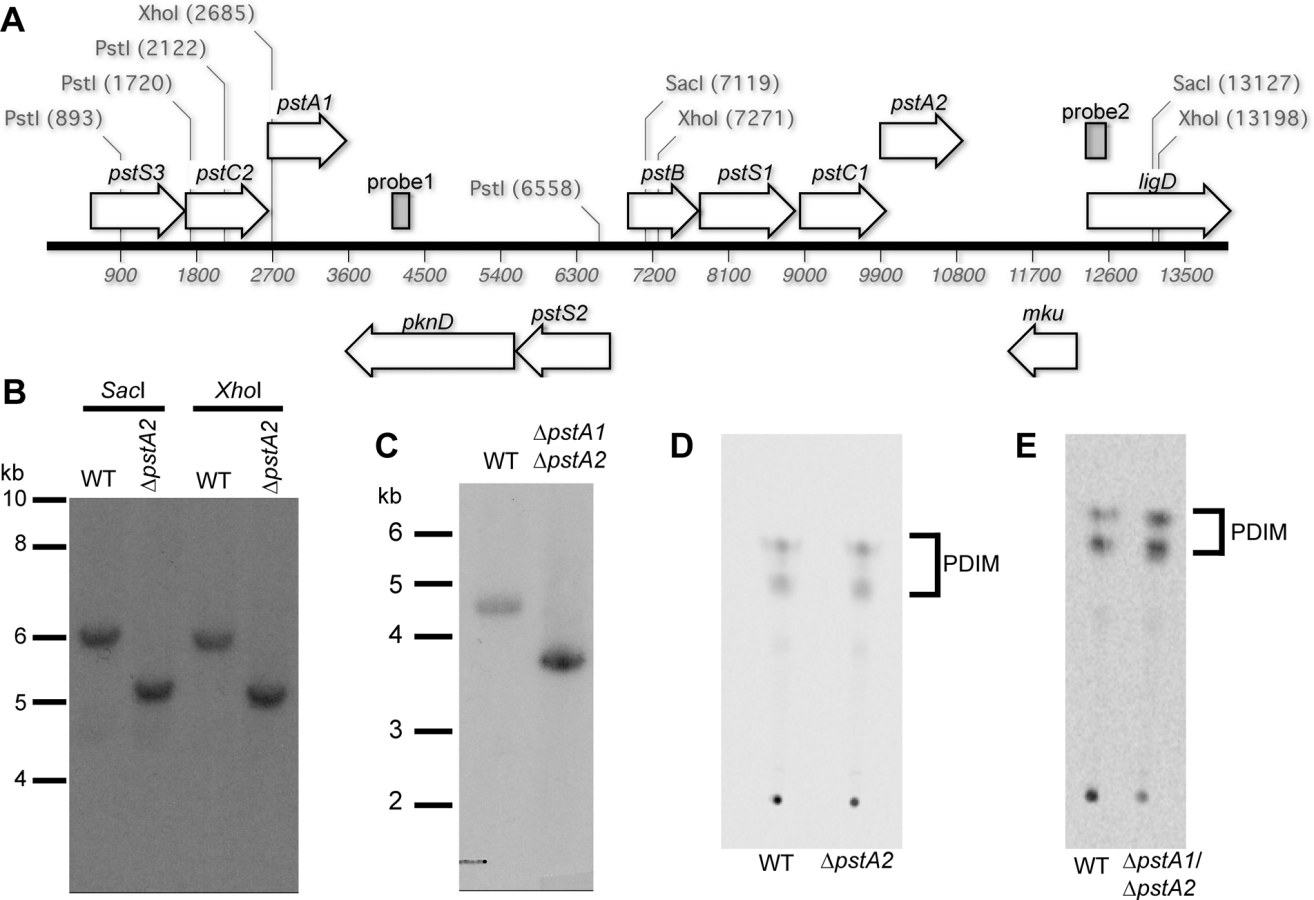
Confirmation of the Δ*pstA2* and Δ*pstA1*Δ*pstA2* deletions by Southern blotting. **(A**) Genetic organization of the *M*. *tuberculosis pst* locus. Genes are indicated by arrows; probes used for Southern blotting are indicated by the gray rectangles. Locations of relevant restriction enzyme sites are shown. (**B**) Southern blot of wild-type Erdman (WT) and Δ*pstA2* genomic DNA digested with either SacI or XhoI, as indicated, and probed with probe2. Molecular weight standards are indicated at the right. The Δ*pstA2* deletion removes 0.9 kb. (**C**) Southern blot of WT and Δ*pstA1*Δ*pstA2* genomic DNA digested with PstI and probed with probe1 to detect the Δ*pstA1* deletion. Molecular weight standards are indicated at the right. The Δ*pstA1* deletion removes 0.89 kb. (**D, E**) Phthiocerol dimycocerosate (PDIM) production by the Δ*pstA2* (**D**) and Δ*pstA1*Δ*pstA2* (**E**) mutants. ^14^C propionate-labeled apolar lipid fractions were extracted and analyzed by thin-layer chromatography. Two spots that correspond to the phthiocerol (methoxy) and phthiodiolone (keto) forms of PDIM are indicated.

To determine if the alternative Pst system, which includes the membrane-spanning component PstA2, is involved in P_i_-responsive gene regulation or virulence, we constructed a deletion of *pstA2* (*rv0936*) by a two-step allelic exchange method in both wild-type (WT) and Δ*pstA1* mutant *M*. *tuberculosis* strains. The *pstA2* deletion was confirmed by PCR and by Southern blotting (Fig 1A–1C). We previously showed that *M*. *tuberculosis* can become deficient for production of phthiocerol dimycocerosate (PDIM), a complex lipid required for full virulence [5], due to spontaneous mutations that arise during routine culture [6]. We therefore confirmed that the Δ*pstA2* and Δ*pstA1*Δ*pstA2* mutant strains are proficient for PDIM production (Fig 1D and 1E).

We previously demonstrated that PstA1 plays a role in regulating gene expression when P_i_ is abundant by controlling activity of the SenX3-RegX3 two-component system [3]. To test whether PstA2 has a similar function in transcriptional regulation, we determined the transcriptome profiles of Δ*pstA2* and Δ*pstA1*Δ*pstA2* bacteria grown in P_i_-rich 7H9 medium using microarrays. The complete gene expression data from these experiments are provided in S1 Table. Under this growth condition, only the *pstA2* gene itself exhibited significantly reduced expression in Δ*pstA2* bacteria compared to the WT control (S2 Table). A total of 69 other genes were differentially expressed greater than two-fold in Δ*pstA2* bacteria compared to WT *M*. *tuberculosis*, but none of these achieved statistical significance (S2 Table). This contrasts with 35 genes that were significantly dysregulated in Δ*pstA1* bacteria grown in the same medium, as determined by reanalysis of previously acquired microarray data [3] using the same normalization and statistical methods (S1 Table). The pattern of gene expression in Δ*pstA1*Δ*pstA2* double mutant bacteria was similar to that previously observed for Δ*pstA1* bacteria (S3 Table). In the Δ*pstA1*Δ*pstA2* strain, 63 genes exhibited significant changes in expression; 30 of these genes were also differentially expressed by Δ*pstA1* bacteria (S3 Table). Differences in gene expression between the Δ*pstA1* and Δ*pstA1*Δ*pstA2* mutant strains observed using the transcriptional profiling approach may be due in part to the stringent cut-offs that were used for selection of significantly regulated genes. Many of the genes that were differentially expressed by Δ*pstA1*Δ*pstA2* bacteria were also dysregulated in Δ*pstA1* bacteria, but did not achieve either the arbitrary > 2-fold ratio or the *P* < 0.05 cut-off (S3 Table). In addition, when gene expression values of the Δ*pstA1* and Δ*pstA1*Δ*pstA2* mutant strains relative to WT were compared, no statistically significant differences between the mutants were observed (S3 Table).

**Fig 2:**
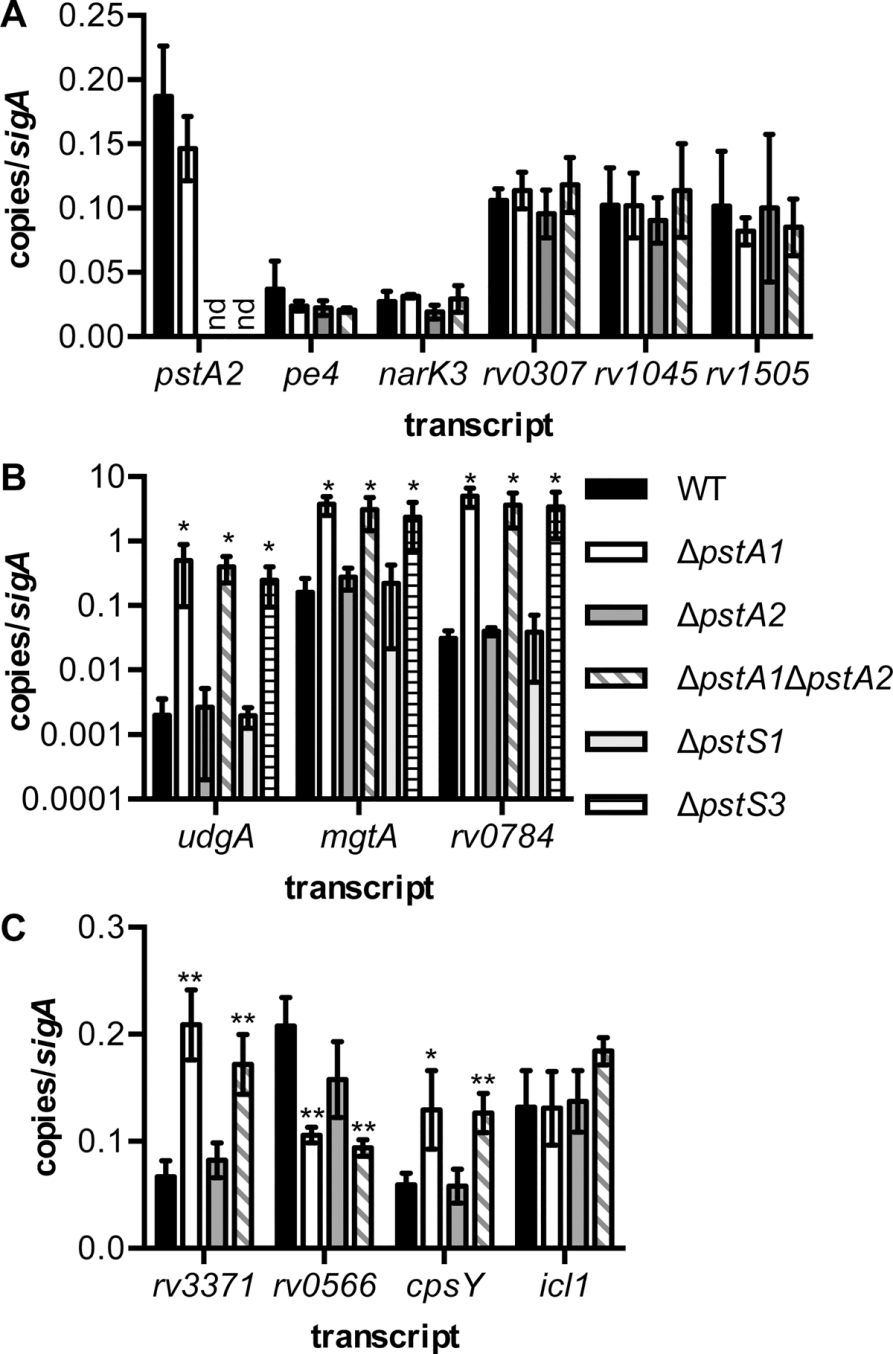
Quantitative RT-PCR analysis of gene expression in *M*. *tuberculosis pst* deletion mutants. RNA was extracted from cultures of *M*. *tuberculosis* WT (black), Δ*pstA1* (white), Δ*pstA2* (dark gray), Δ*pstA1*Δ*pstA2* (diagonal stripes), Δ*pstS1* (light gray), and Δ*pstS3* (horizontal stripes) bacteria grown to mid-exponential phase (OD_600_ of 0.5) in P_i_-rich 7H9 medium. Transcript abundance relative to abundance of *sigA* was determined by real-time quantitative RT-PCR. Data shown are the mean ± standard deviation of three independent experiments. nd indicates transcripts that were undetectable in Δ*pstA2* and Δ*pstA1*Δ*pstA2* bacteria. Asterisks indicate statistically significant differences from the WT control: *P < 0.05, **P < 0.005. **(A)** Validation of transcriptional profiling of Δ*pstA2* bacteria. **(B)** Transcripts that are differentially expressed in Δ*pstA1* bacteria in a RegX3-dependent manner. **(C)** Transcripts predicted by microarray analysis to be differentially expressed by Δ*pstA1*Δ*pstA2* bacteria.

To validate the results of the transcriptional profiling, we performed quantitative RT-PCR (qRT-PCR) experiments. To confirm that deletion of *pstA2* did not result in any reproducible changes in transcription, we examined expression of *pstA2* itself and five other genes that were differentially expressed near the 2-fold cut-off but that did not achieve statistical significance. The *pstA2* transcript was undetectable by qRT-PCR in both the Δ*pstA2* and Δ*pstA1*Δ*pstA2* mutants, in which the entire *pstA2* gene has been deleted (Fig 2A). We did not detect any significant change in expression of the *pe4*, *narK3*, *rv0307*, *rv1405*, and *rv1505* transcripts by qRT-PCR, confirming the transcriptional profiling analysis (Fig 2A). Next, we examined expression of several genes that we previously demonstrated were differentially expressed by Δ*pstA1* bacteria [3]. The expression of each of these genes (*udgA*, *mgtA*, and *rv0784*) was unchanged by the Δ*pstA2* mutation (Fig 2B). In addition, each of these genes was expressed at a significantly higher level in Δ*pstA1* and Δ*pstA1*Δ*pstA2* bacteria compared to the level in the WT control (Fig 2B, *P* < 0.05).

Finally, we performed qRT-PCR experiments to validate the Δ*pstA1*Δ*pstA2* transcriptional profiling. The genes we selected for further analysis include two that were significantly differentially expressed in the Δ*pstA1*Δ*pstA2* double mutant but not the Δ*pstA1* single mutant (*rv3371* and *rv0556*, S3 Table). We also analyzed expression of two genes that were differentially expressed above the arbitrary 2-fold cut-off in the Δ*pstA1*Δ*pstA2* mutant, but that did not achieve the *P* < 0.05 significance cut-off (*cpsY* and *icl1*, S1 Table). There were no significant changes in expression of these four genes in Δ*pstA2* bacteria, consistent with the microarray data (Fig 2C). The qRT-PCR data did not, however, fully corroborate the transcriptional profiling data for the Δ*pstA1* and Δ*pstA1*Δ*pstA2* mutants. Both *rv3371* and *rv0566* were significantly dysregulated in Δ*pstA1*Δ*pstA2* bacteria, as predicted by the microarray analysis, but these genes were also significantly dysregulated in Δ*pstA1* bacteria (Fig 2C). These data suggest that many of the genes we identified with significant differential expression in the Δ*pstA1*Δ*pstA2* double mutant are also differentially expressed in the Δ*pstA1* mutant. The *cpsY* gene was also significantly over-expressed in both the Δ*pstA1* and Δ*pstA1*Δ*pstA2* mutants (Fig 2C). In contrast, *icl1* showed no significant change in expression in either mutant (Fig 2C). These data suggest that the transcriptional profiling data were analyzed with a stringent statistical significance cut-off that may have excluded some differentially expressed genes. Since the Δ*pstA2* mutation did not cause any significant changes in gene expression, either alone or in combination with the Δ*pstA1* mutation, our data suggest that PstA2 does not have a role in negative regulation of gene expression in response to sufficient extracellular P_i_.

### PstA2 Is Not Required for Replication or Virulence in Aerosol-Infected Mice

**Fig 3:**
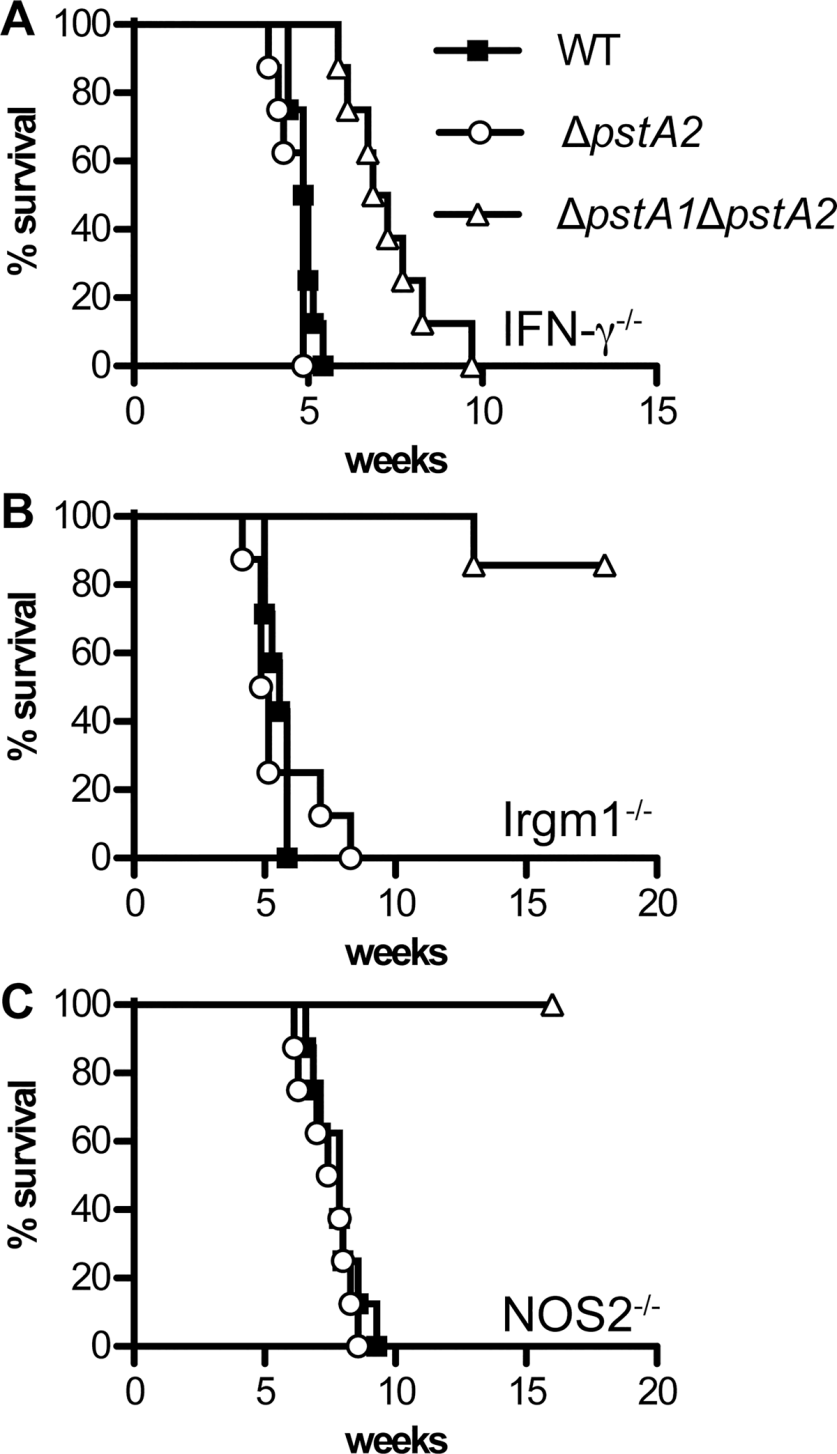
Virulence of *M*. *tuberculosis* Δ*pstA2* and Δ*pstA1*Δ*pstA2* mutants in immune-deficient mice. Groups of immune-deficient mice (n = 8), all on the C57BL/6 background, were aerosol-infected with ~100 CFU of wild-type *M*. *tuberculosis* (WT, squares), Δ*pstA2* (circles), or Δ*pstA1*Δ*pstA2* (triangles) and monitored for signs of illness. Moribund mice were euthanized and scored as dead. **(A**) IFN-γ^-/-^, (**B**) Irgm1^-/-^, (**C**) NOS2^-/-^.

**Fig 4:**
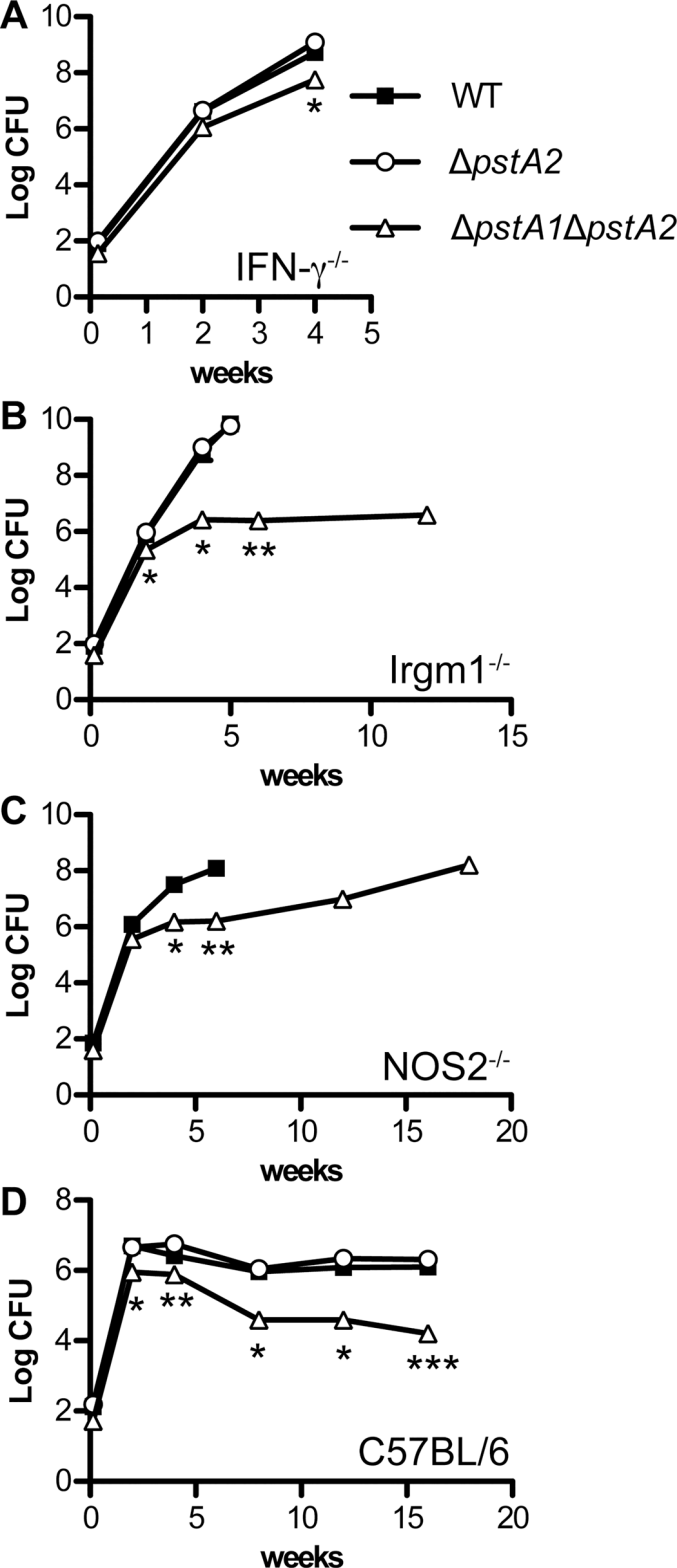
Growth kinetics of *M*. *tuberculosis* Δ*pstA2* and Δ*pstA1*Δ*pstA2* mutants in aerosol-infected mice. Mice were aerosol-infected with ~100 CFU of *M*. *tuberculosis* WT (squares), Δ*pstA2* (circles), or Δ*pstA1*Δ*pstA2* (triangles). Groups of mice (n = 4) were sacrificed at the indicated time points. Bacterial CFU were enumerated by plating serially diluted lung homogenates on 7H10 agar and incubating 3–4 weeks at 37°C. All mouse strains were on the C57BL/6 background. Symbols represent means; error bars indicate standard error of the mean. Asterisks indicate statistically significant differences between WT and Δ*pstA1*Δ*pstA2*: *P < 0.05, **P < 0.005, ***P < 0.0005. No significant differences in pulmonary CFU were observed between the WT and Δ*pstA2* strains. (**A**) IFN-γ^-/-^, (**B**) Irgm1^-/-^, (**C**) NOS2^-/-^, (**D**) C57BL/6.

We recently demonstrated that *M*. *tuberculosis* PstA1 is dispensable for replication and virulence in IFN-γ-deficient (IFN-γ^-/-^) mice, but is required for sustained replication and virulence in mice lacking either the IFN-γ-inducible nitric oxide synthase (NOS2^-/-^) or the IFN-γ-regulated GTPase Irgm1 (Irgm1^-/-^) [3]. To test if PstA2 is similarly required for virulence in these immune-deficient mice, IFN-γ^-/-^, NOS2^-/-^, and Irgm1^-/-^ mice were infected by the aerosol route with approximately 100 colony-forming units (CFU) *M*. *tuberculosis* wild-type (WT) or Δ*pstA2* strains and survival time was monitored. In contrast to the Δ*pstA1* mutant, Δ*pstA2* bacteria were fully virulent in all three strains of immune-deficient mice (Fig 3). Survival of mice infected with the Δ*pstA2* mutant was not significantly different from survival of mice infected with WT bacteria. Consistent with these data, deletion of *pstA2* had no impact on the ability of *M*. *tuberculosis* to replicate in the lungs of IFN-γ^-/-^ or Irgm1^-/-^ mice (Fig 4A and 4B). Pulmonary CFU of the Δ*pstA2* mutant were not significantly different from the WT control at any time point examined. These data suggest that PstA2 alone is not required for replication or virulence in immune-deficient mice. To extend these results, wild-type C57BL/6 mice were infected by the aerosol route with the *M*. *tuberculosis* WT or Δ*pstA2* strains and pulmonary CFU were monitored. Δ*pstA2* bacteria replicated in the lungs of C57BL/6 mice with kinetics identical to WT bacteria, and persisted equally well during the chronic phase of the infection (Fig 4D). These data suggest that PstA2 is not required for *M*. *tuberculosis* replication or persistence in a murine aerosol infection model.

### PstA2 Cooperates with PstA1 to Resist Innate Immune Defenses Independent of IFN-γ

Although PstA2 alone is not essential for *M*. *tuberculosis* replication or virulence in mice, we hypothesized that the two Pst systems might exhibit functional redundancy, since they are both predicted to be involved in P_i_ uptake. To test this possibility, we examined replication and virulence of Δ*pstA1*Δ*pstA2* double mutant bacteria in aerosol-infected immune-deficient and wild-type C57BL/6 mice. Generally, the Δ*pstA1*Δ*pstA2* double mutant behaved similarly to the Δ*pstA1* single mutant. Δ*pstA1*Δ*pstA2* bacteria were significantly attenuated in both Irgm1^-/-^ (Fig 3B) and NOS2^-/-^ mice (Fig 3C) as assessed by survival of the infected animals. Mean survival time of Irgm1^-/-^ mice infected with WT bacteria was 39 days, while the majority of mice infected with Δ*pstA1*Δ*pstA2* bacteria survived 126 days (18 weeks), when the experiment was terminated (Fig 3B, *P* = 0.0003). Similarly, the mean survival time of NOS2^-/-^ mice infected with WT bacteria was 55 days, while all mice infected with the Δ*pstA1*Δ*pstA2* mutant survived 112 days (16 weeks) when the experiment was terminated (Fig 3C, *P* = 0.0024). The Δ*pstA1*Δ*pstA2* strain was defective for sustained replication in the lungs of either Irgm1^-/-^ (Fig 4B) or NOS2^-/-^ (Fig 4C) mice, similar to the Δ*pstA1* mutant [3].

In contrast to Δ*pstA1* bacteria that were fully virulent in IFN-γ^-/-^ mice [3], the Δ*pstA1*Δ*pstA2* mutant was attenuated in IFN-γ^-/-^ mice (Figs 3A and 4A). In two independent experiments, IFN-γ^-/-^ mice infected with Δ*pstA1*Δ*pstA2* bacteria survived significantly longer than those infected with WT *M*. *tuberculosis*, with an increase in the mean survival time of at least 15 days (Fig 3A, *P* < 0.0001 in both experiments). Pulmonary CFU of Δ*pstA1*Δ*pstA2* bacteria in IFN-γ^-/-^ mice were also significantly reduced compared to the WT control at four weeks post-infection (Fig 4A, *P* = 0.04). These results suggest that Δ*pstA1*Δ*pstA2* bacteria are sensitive to an innate immune response that is independent of the IFN-γ cytokine. Consistent with this observation, Δ*pstA1*Δ*pstA2* bacteria exhibited a replication defect in the lungs of wild-type C57BL/6 mice during the acute phase of infection (Fig 4D). Pulmonary CFU of the Δ*pstA1*Δ*pstA2* mutant were significantly different from the WT control at two weeks post-infection (*P* = 0.0003). Δ*pstA1*Δ*pstA2* bacteria also displayed a persistence defect that was similar to the phenotype we previously observed for the Δ*pstA1* mutant (Fig 4D). However, at each time point, CFU of the Δ*pstA1*Δ*pstA2* mutant were reduced approximately 4-fold compared to the Δ*pstA1* single mutant [3]. These data suggest that the alternative P_i_ uptake system that includes PstA2 is required for survival of innate immune responses independent of IFN-γ and for persistence when the P_i_ uptake system that includes PstA1 is non-functional.

Despite repeated efforts to achieve similar input doses, Δ*pstA1*Δ*pstA2* bacteria consistently failed to colonize the lungs of aerosol-infected mice at the same level as WT *M*. *tuberculosis*. In experiments examining *M*. *tuberculosis* replication and virulence in IFN-γ^-/-^ mice, for example, only 36 or 38 CFU of Δ*pstA1*Δ*pstA2* bacteria were introduced to the lungs, compared to 80 or 96 CFU of the WT strain. We tested if Δ*pstA1*Δ*pstA2* bacteria are sensitive to aerosolization by examining viability of the inoculum before and immediately after the infection procedure. While viability of WT *M*. *tuberculosis* was not altered by aerosolization, viability of the Δ*pstA1*Δ*pstA2* mutant was reduced by as much as 60% (data not shown). We therefore cannot exclude the possibility that the attenuation of Δ*pstA1*Δ*pstA2* bacteria may be attributable to a reduced input dose, due to susceptibility either to stress experienced during nebulization or to innate immune responses encountered during the first 24 hours of infection.

### Deletion of *pstA2* Enhances Resistance to Acidic pH

**Fig 5:**
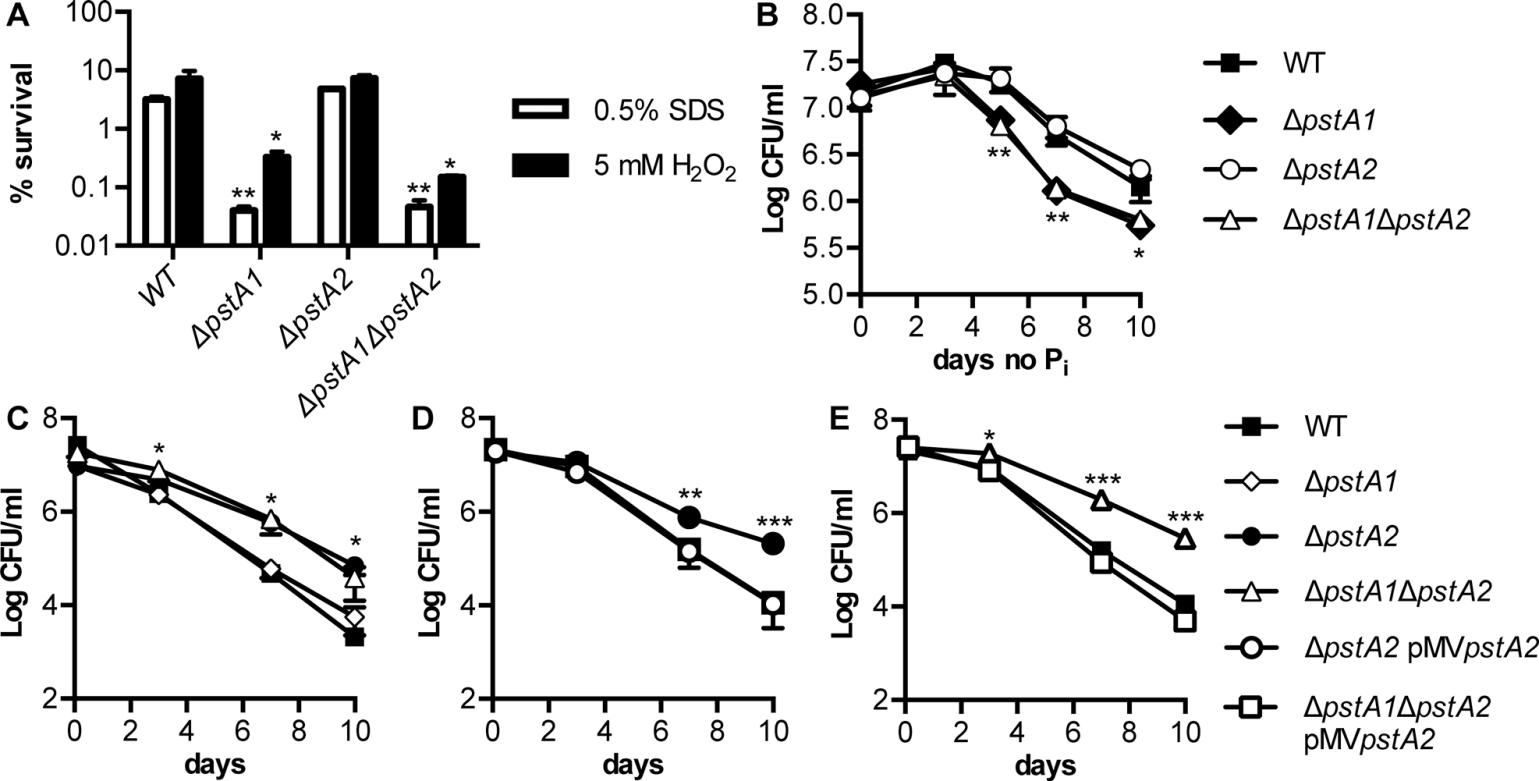
Deletion of *pstA2* causes increased resistance to acidified medium. *M*. *tuberculosis* WT, Δ*pstA1*, Δ*pstA2*, Δ*pstA1*Δ*pstA2*, Δ*pstA2* pMV*pstA2* and Δ*pstA1*Δ*pstA2* pMV*pstA2* strains were grown to mid-exponential phase in P_i_-rich 7H9 medium and then subjected to stress conditions, as indicated. Bacterial CFU were enumerated by plating serially diluted cultures on 7H10 agar and incubating for 3 to 4 weeks at 37°C. Percent survival was calculated as (CFU poststress)/(CFU pre-stress)x100. Data shown are means ± standard deviations of three independent cultures from one experiment, and are representative of at least three independent experiments. Asterisks indicate statistically significant differences between the deletion mutant and WT control for panels A-C or between the deletion mutant and both the WT and complemented strains for panels D and E: *P < 0.05, **P < 0.005, ***P < 0.0005. **(A)** Cell wall stress (sodium dodecyl sulfate, SDS) and reactive oxygen stress (hydrogen peroxide, H_2_O_2_). Either 0.5% SDS or 5 mM H_2_O_2_ was added to each culture. CFU were enumerated at 0 and 24 hours and percent survival was calculated. **(B)** P_i_ starvation. Bacteria were shifted to P_i_-free medium and CFU were enumerated at 0, 3, 5, 7, and 10 days. **(C-E)** Acidic pH stress. Bacteria were shifted to acidified 7H9 medium pH 4.5 containing 0.1% Tween-80 and CFU were enumerated at 0, 3, 7, and 10 days.

We previously showed that Δ*pstA1* bacteria are sensitive to a variety of stress conditions *in vitro*, including exposure to detergents and reactive oxygen species [3]. Sensitivity to each of these stress conditions was attributed to aberrant gene expression mediated by the response regulator RegX3, since stress sensitivity was suppressed by deletion of the *regX3* gene [3]. To determine if PstA2 might be similarly required for stress resistance, we tested sensitivity of Δ*pstA2* and Δ*pstA1*Δ*pstA2* bacteria to a variety of stress conditions, including some to which the Δ*pstA1* mutant was fully resistant. *M*. *tuberculosis* Δ*pstA2* bacteria were equally resistant as the WT control to both the detergent sodium dodecyl sulfate (SDS) and the reactive oxygen species hydrogen peroxide (H_2_O_2_) (Fig 5A). In addition, Δ*pstA1*Δ*pstA2* bacteria were no more sensitive to either of these stress conditions than the Δ*pstA1* mutant (Fig 5A). Similarly, Δ*pstA2* bacteria persisted equally well during P_i_ starvation as WT *M*. *tuberculosis*, and Δ*pstA1*Δ*pstA2* bacteria were no more sensitive to P_i_ starvation than the Δ*pstA1* mutant (Fig 5B).

Surprisingly, Δ*pstA2* bacteria were significantly more resistant than the WT control to acidified 7H9 medium, pH 4.5 (Fig 5C). Increased resistance of Δ*pstA2* bacteria to acid was independent of the presence or absence of the Δ*pstA1* mutation, since the Δ*pstA1*Δ*pstA2* mutant also exhibited increased acid resistance (Fig 5C). We confirmed that the increased resistance to acid was due to the deletion of *pstA2* by complementation. Strains harboring the pMV*pstA2* complementing plasmid were equally sensitive as the WT control to acidified 7H9 medium (Fig 5D and 5E).

### Sensitivity to Acidic pH Is Conferred by the Alternative Pst System

To determine if sensitivity to acidic pH is caused by expression of the alternative Pst P_i_ uptake system, or is solely dependent on PstA2, we tested mutants lacking the PstS substrate binding proteins of the Pst system for acid sensitivity. PstS3 is encoded in the same operon as PstA1 [7] and is likely to operate as a component of this primary P_i_ uptake system. PstS1 is encoded in the same operon as PstA2 [7] and may therefore participate in this alternative P_i_ uptake system. We generated in-frame unmarked deletions of *pstS3* and *pstS1* and confirmed the deletions by PCR. To test if either of these PstS substrate binding proteins is required for negative regulation of RegX3-dependent genes in P_i_-rich medium, we examined expression of the *udgA*, *mgtA*, and *rv0784* genes by qRT-PCR. Levels of the *udgA*, *mgtA*, and *rv0784* transcripts were not altered by the Δ*pstS1* mutation, but were significantly increased in Δ*pstS3* bacteria (Fig 2B). These data suggest that only PstS3 interacts with the Pst system that includes PstA1 and that participates in regulation of gene expression in response to P_i_ availability.

**Fig 6:**
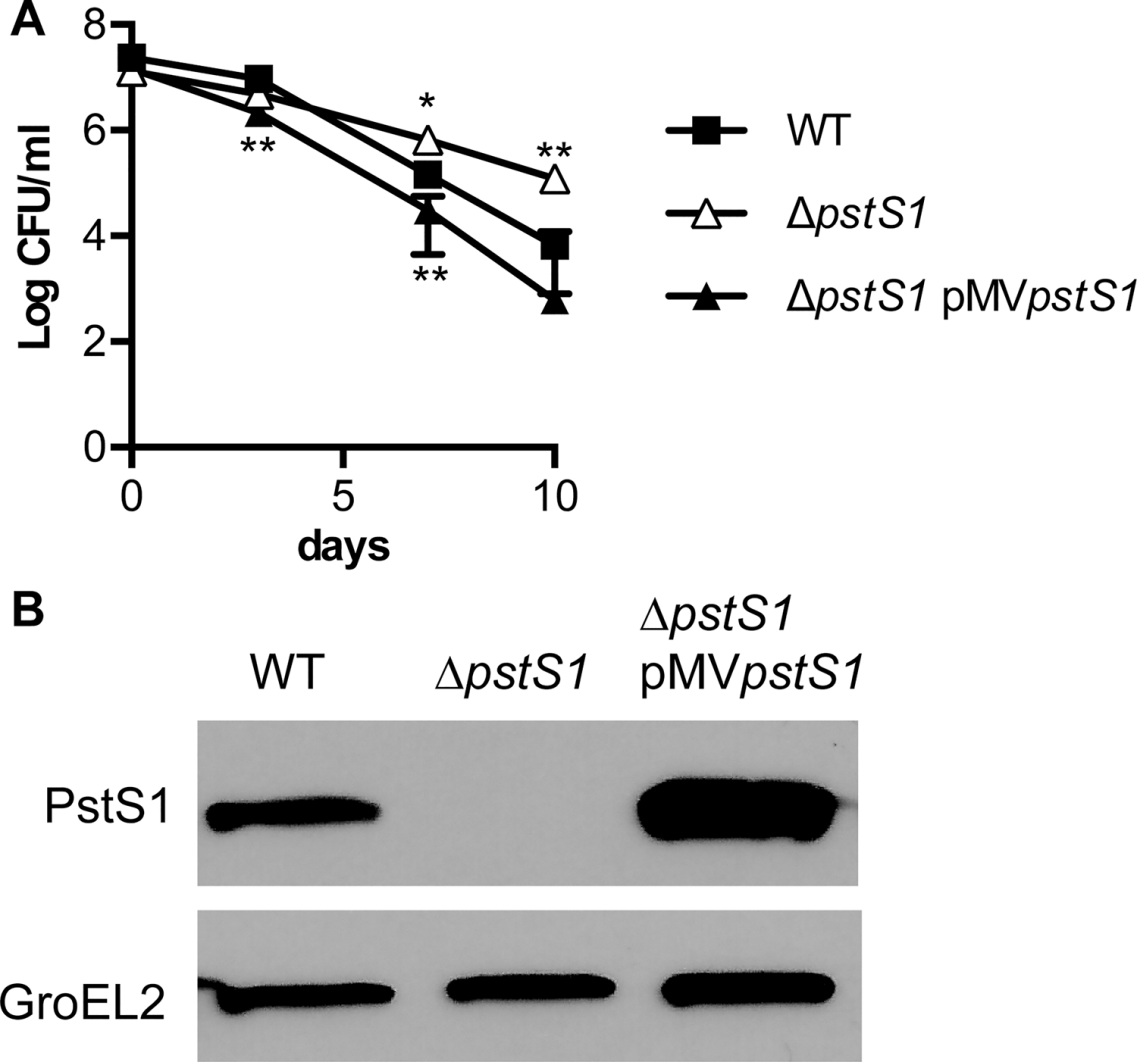
Deletion of *pstS1* causes increased resistance to acidified medium. (**A**) *M*. *tuberculosis* WT, Δ*pstS1*, and Δ*pstS1* pMV*pstS1* were grown to mid-exponential phase in P_i_-rich 7H9 medium and then shifted to acidified 7H9 medium pH 4.5 containing 0.1% Tween-80. Bacterial CFU were enumerated on days 0, 3, 7, and 10 by plating serially diluted cultures on 7H10 agar and incubating for 3 to 4 weeks at 37°C. Data shown are means ± standard deviations of three independent cultures from one experiment and are representative of at least three independent experiments. Asterisks indicate statistically significant differences from the WT control: *P < 0.05, **P < 0.005, ***P < 0.0005. (**B**) PstS1 protein production. Whole cell lysate proteins (4 μg) of WT, Δ*pstS1*, Δ*pstS1* pMV*pstS1* were subjected to SDS-PAGE and Western blot analysis. Mouse monoclonal anti-PstS1 and anti-GroEL2 antibodies were used to detect PstS1 and GroEL2, a cell-associated protein that serves as a loading control. Results shown are representative of two independent experiments.

We subsequently tested these Δ*pstS3* and Δ*pstS1* deletion mutants for survival in acidified 7H9 medium, pH4.5. Like the Δ*pstA1* mutant, the Δ*pstS3* mutant was equally sensitive as the WT control to acidified medium (data not shown). In contrast, the Δ*pstS1* mutant was more resistant to 7H9 medium pH 4.5 (Fig 6A). To determine if the increased resistance to acidic pH was due to deletion of *pstS1*, we complemented the mutation by providing a wild-type copy of *pstS1* on an episomal plasmid. The strain harboring pMV*pstS1* plasmid was significantly more sensitive to acidified 7H9 medium than the WT control, demonstrating that *pstS1* is responsible for sensitivity to acidic pH (Fig 6A). We tested if the hyper-sensitivity of the Δ*pstS1* pMV*pstS1* strain to acidified medium is due to over-expression of PstS1 from the complementing plasmid by performing Western blotting experiments. Production of PstS1 was increased in whole cell extracts from the Δ*pstS1* pMV*pstS1* strain compared to the WT control (Fig 6B). These experiments also demonstrated that PstS1 is absent from Δ*pstS1* mutant bacteria (Fig 6B). These data suggest that sensitivity to acidic pH is caused by expression of an alternative Pst P_i_ uptake system that includes both PstA2 and PstS1 and is intrinsic to the transporter itself.

## Discussion

Many bacteria require the Pst P_i_ uptake system both for survival of P_i_ starvation and for virulence [8]. *M*. *tuberculosis* is unusual because its genome encodes two complete Pst systems [4]. It is likely that each of these systems has some function for the bacterium, since both have been maintained during evolution. However, it was unclear if the two Pst systems have unique or partially redundant functions. We previously showed that the Pst system that includes the transmembrane component PstA1 is required both for virulence in a murine aerosol infection model, and for regulation of gene expression in response to the external P_i_ concentration [3]. Here, we examined if the alternative Pst P_i_ uptake system, which includes the transmembrane component PstA2, is necessary for *M*. *tuberculosis* virulence. Our data demonstrate that the alternative Pst P_i_ uptake system component PstA2 is not required either for replication in the lungs of aerosol-infected mice or for virulence. Our results are consistent with data from several genome-wide screens that identified PstA1 or its cognate substrate binding protein PstS3, but not PstA2 or PstS1, as potential *M*. *tuberculosis* virulence factors [9–11]. We further demonstrate that PstA2 does not participate in regulation of gene expression during growth in P_i_-rich medium. Our data suggest that the gene regulation and virulence functions are unique to the Pst system that includes PstA1.

Although PstA2 is not required for replication or virulence in a murine aerosol infection model, it is necessary for survival of innate immune responses in the absence of PstA1 function. In IFN-γ^-/-^ mice, the Δ*pstA1*Δ*pstA2* mutant exhibited a reduced replication rate in the lungs and significantly reduced virulence. These results suggest that the two *M*. *tuberculosis* Pst systems have partially redundant functions. It is possible that both Pst systems contribute to P_i_ scavenging, and that *M*. *tuberculosis* requires the activity of at least one Pst system to survive P_i_ limitation encountered in the lungs. The Δ*pstA1*Δ*pstA2* double mutant was attenuated in IFN-γ^-/-^ mice, suggesting that P_i_ limitation is independent of macrophage activation by this cytokine. However, the Δ*pstA1*Δ*pstA2* double mutant is still capable of replicating in the lungs during the acute phase of infection, suggesting that some bacteria are present in a tissue environment that contains either sufficient P_i_ for acquisition by one of the relatively low-affinity Pit transporters, or sufficient organic phosphate sources (e.g. glycerol phosphates). The increased attenuation of Δ*pstA1*Δ*pstA2* bacteria relative to the Δ*pstA1* mutant may also be due to a decreased infectious dose. Despite multiple attempts, we were not able to obtain an equivalent input dose of the Δ*pstA1*Δ*pstA2* mutant, though both the Δ*pstA2* and Δ*pstA1* single mutants were fully proficient for colonizing aerosol-infected mice at the ~100 CFU input dose [3]. It is possible that the double mutant is sensitive to the aerosolization procedure, or that it is more readily killed by alveolar macrophages during the first 24 hours of infection. Finally, since we did not conduct complementation analysis, it is possible that an off-target secondary mutation contributes to the additional sensitivity of the Δ*pstA1*Δ*pstA2* mutant to innate immune responses.

We additionally demonstrate that the gene regulation function of the Pst system in *M*. *tuberculosis* is unique to the transporter that includes PstA1 and PstS3. Deletion of either *pstA1* or *pstS3* led to constitutive expression of known RegX3-regulated genes, while deletion of either *pstA2* or *pstS1* did not alter expression of these genes. It is possible that only the Pst system that includes PstA1 and PstS3, and not the system that includes PstA2 and PstS1, is capable of interacting with the SenX3-RegX3 two-component signal transduction system that directly regulates transcription. Indeed, there are substantial differences between the sequences of these Pst system components. PstA1 and PstA2 only exhibit 43% sequence similarity (28.8% identical), while PstS3 and PstS1 are only 36% similar (27.5% identical). It is likely that these sequence differences determine the ability to participate in signal transduction with SenX3-RegX3. Transcriptome analysis of the Δ*pstA1*Δ*pstA2* mutant revealed additional genes that may be included in the RegX3 regulon that were not uncovered in our previous analysis of the Δ*pstA1* mutant. This expands our knowledge of genes that are likely to be regulated by RegX3 in response to P_i_ availability.

Our data demonstrating that PstA2 is dispensable for *M*. *tuberculosis* replication in the mouse aerosol infection model contrast with a previous report that suggested PstS1, a putative substrate-binding proteins of the alternative Pst P_i_ uptake system, is necessary for replication in intravenously infected mice [12]. Differences in the infection route may account for the differences in results. The *pstS1* mutant bacteria that were previously analyzed might also harbor a secondary mutation, such as a mutation affecting PDIM biosynthesis, which causes reduced replication in the lungs and spleen. Since complementation analysis was not performed in this previous study we cannot rule out this possibility. Finally, PstS1 may have a virulence function that is separable from its role in P_i_ uptake, as it was recently suggested to promote macrophage phagocytosis by binding the mannose receptor [13].

Unexpectedly, the Δ*pstA2* and Δ*pstA1*Δ*pstA2* mutants were more resistant to acidified 7H9 medium. We observed similar enhanced resistance to acid in a Δ*pstS1* mutant, suggesting that this phenotype is attributable to expression of the transporter itself. Since the Δ*pstA2* mutation did not influence gene expression, the acid resistance phenotype may be due to the P_i_ uptake function of this alternative Pst system. The crystal structure of the PstS1 phosphate-binding protein of *M*. *tuberculosis* was previously determined [14]. The phosphate-binding site in PstS1 contains two aspartate residues, suggesting that it might preferentially coordinate the monobasic form of phosphate (H_2_PO_4_^-^) that predominates phosphate solutions at pH values below the pK_2_ (7.21) [14]. PstS3 contains only one aspartate residue within its phosphate-binding site, suggesting that it primarily coordinates the dibasic form of phosphate (HPO_4_^2-^) [15]. Although purified *M*. *tuberculosis* PstS1 did not exhibit an increased affinity for P_i_ at low pH *in vitro* [16], it is possible that *in vivo* PstS1 is required for transport of P_i_ when the bacteria encounter acidic conditions. Under acidic conditions WT bacteria might readily transport the monobasic form of phosphate, along with the additional proton, via the P_i_ uptake system composed of PstS1 and PstA2, leading to acidification of the cytoplasm. In contrast, Δ*pstA2* and Δ*pstS1* bacteria would be unable to transport the protonated form of phosphate, leading to reduced accumulation of protons in the cytoplasm, and increased resistance to acid stress. Alternatively, the Δ*pstA2* and Δ*pstS1* mutants may be more resistant to free fatty acids that are released by hydrolysis of the Tween-80 or albumin components of the acidified culture medium [17]. Additional work will be required to distinguish between these possibilities and to determine whether pH influences the P_i_ transport activity of either *M*. *tuberculosis* Pst system.

Although our data indicate that the Pst system including PstS1 and PstA2 has some physiological function that influences sensitivity to acid, it is unclear at this time why *M*. *tuberculosis* has maintained two independent Pst P_i_ uptake systems. Other slow-growing mycobacteria, including *M*. *bovis* and *M*. *avium*, have also maintained two complete Pst systems in their genomes. In addition, the fast-growing *M*. *smegmatis* uses both Pst and Phn, two high-affinity P_i_ transporters, for P_i_ uptake [18]. Given that both Pst systems have been maintained during *M*. *tuberculosis* evolution, it is likely that the alternative system including PstA2 is necessary for some aspect of tuberculosis pathogenesis that is not well modeled in the mouse. PstA2 may be required for *M*. *tuberculosis* transmission. Alternatively, PstA2 may be required for P_i_ uptake when the bacteria are located in hypoxic or necrotic granulomas, which do not develop in the C57BL/6 mouse model.

In *M*. *tuberculosis* and other mycobacteria, it is possible that both Pst systems are involved in P_i_ uptake, but that they have different P_i_ affinities. In *Vibrio cholerae*, another organism with two Pst systems, the alternative Pst2 system is induced in biofilms and contributes to survival of the bacteria in P_i_-limited environments after biofilm dispersal, likely by enhancing their ability to scavenge P_i_ [19]. *Halobacterium salinarum* also has two Pst P_i_ uptake systems: one is constitutively expressed and has a lower affinity for P_i_ while the other has a higher affinity and is induced by P_i_ starvation [20]. In *M*. *tuberculosis*, only the operon encoding PstS3 and PstA1, and not the operon encoding PstS1 and PstA2, is induced during P_i_ limitation [21]. It is possible that the system including PstS3 and PstA1 has a higher affinity for P_i_, and is induced to enable more efficient P_i_ scavenging during P_i_ limitation. Alternatively, there could be specialization of function of the *M*. *tuberculosis* Pst systems, with the system including PstA1 being specifically involved in P_i_ sensing and gene regulation, while the alternative Pst system including PstA2 is specialized for P_i_ uptake. Further work examining the role of each *M*. *tuberculosis* Pst system in P_i_ uptake under various conditions will be required to distinguish between these possibilities.

## Materials and Methods

### Bacterial Culture Conditions

*M*. *tuberculosis* Erdman and derivative strains were grown at 37°C in Middlebrook 7H9 (Difco) liquid culture medium supplemented with 10% albumin-dextrose-saline (ADS), 0.5% glycerol, and 0.1% Tween-80 or on Middlebrook 7H10 (Difco) solid culture medium supplemented with 10% OADC (BD Biosciences) and 0.5% glycerol. Frozen stocks were prepared by growing liquid cultures to mid-exponential phase (OD_600_ 0.6 to 0.8), adding glycerol to 15% final concentration, and storing aliquots at -80°C. For acidic 7H9 medium, complete 7H9 (with supplements) was adjusted to pH 4.5 with hydrochloric acid prior to filter sterilization. For phosphate-free (P_i_-free) 7H9 broth, a 100 liquid stock of reconstituted P_i_-free 7H9 base was prepared. 1× P_i_-free 7H9 was made with 0.5% glycerol, 10% ADS, 0.1% Tween-80, and 50 mM MOPS buffer, pH 6.6.

### Cloning

Constructs for deletion of *pstA2*, *pstS1* or *pstS3* were generated in the allelic exchange vectors pJG1100 [6] or pJG1111b [22]. pJG1100 contains the *aph* (kanamycin resistance), *hyg* (hygromycin resistance), and *sacB* (sucrose sensitivity) markers; pJG1111b additionally contains *lacZ* (**β-**galactosidase) to enable blue-white screening. Genomic regions 500–800 bp upstream and downstream of the genes to be deleted were PCR amplified from *M*. *tuberculosis* Erdman genomic DNA using the oligonucleotides listed in S4 Table. For Δ*pstA2*, the upstream and downstream regions of homology were joined together by splicing by overlap extension PCR [23], cloned in pCR2.1-TOPO (Invitrogen), and sequenced. The Δ*pstA2* insert was removed from pCR2.1 by digestion with PacI/AscI and ligated to similarly digested pJG1100 to create pAN012. This Δ*pstA2* allelic exchange construct encodes the first 2 amino acids of PstA2 fused to the final amino acid of PstA2. For the Δ*pstS1* and Δ*pstS3* constructs, the reverse primer for amplification of the upstream region was designed with an AvrII restriction site in-frame with the translation start codon; the corresponding forward primer for amplification of the downstream region was designed with an AvrII restriction site in-frame with the stop codon. The upstream and downstream PCR products were cloned in pCR2.1-TOPO (Invitrogen) and sequenced. The Δ*pstS1* or Δ*pstS3* upstream and downstream regions were removed from pCR2.1 by restriction with PacI/AvrII and AvrII/AscI, respectively, and ligated together in pJG1100 (Δ*pstS3*) or pJG1111b (Δ*pstS1*) between the PacI and AscI sites. The Δ*pstS1* construct pMK202 encodes the first 3 amino acids of PstS1 fused to the last 2 amino acids of PstS1. The Δ*pstS3* construct pAT215 encodes the first 3 amino acids of PstS3 fused to the last 2 amino acids of PstS3.

Vectors for complementation of the Δ*pstA2* or the Δ*pstS1* deletions were constructed in the episomal plasmid pMV261. The putative promoter region upstream of *pstB* and the first 5 codons of *pstB* were amplified with primers A2P1F and A2P1R (S4 Table). The last 48 codons of *pstC1* and the complete *pstA2* gene were amplified with primers C1A2F and C1A2R (S4 Table). The resulting PCR products were cloned in pCR8-TOPO (Invitrogen) and sequenced. Inserts were removed by restriction with PstI/BglII and BglII/SalI, respectively, and ligated together in PstI/SalI digested pMV261 to generate pMV*pstA2*. The PstI/SalI digest removes the *hsp60* promoter from pMV261, so that in pMV*pstA2* expression of *pstA2* is controlled by the *pstB* promoter. For complementation of Δ*pstS1*, the putative *pstB* promoter was amplified by PCR with primers ProBF and ProBR; full-length *pstS1* was amplified with primers S1CF2 and S1CR by PCR (S4 Table). Both PCR products were cloned in pCR2.1-TOPO and sequenced. The *pstB* promoter and *pstS1* inserts were restricted with XbaI/EcoRI and EcoRI/HindIII, respectively, and ligated to pMV261 restricted with XbaI and HindIII. The XbaI/HindIII digest removes the *hsp60* promoter from pMV261, thereby placing *pstS1* under control of the *pstB* promoter.

### Strain Construction

*M*. *tuberculosis* strains harboring unmarked in-frame Δ*pstA2*, Δ*pstS1*, or Δ*pstS3* deletions were constructed by a two-step homologous recombination method for allelic exchange [24, 25] using the vectors pAN012, pMK202, or pAT215, respectively. These plasmids were introduced into wild-type *M*. *tuberculosis* Erdman by electroporation as described [3]. The Δ*pstA1*Δ*pstA2* double deletion mutant was generated by electroporating the Δ*pstA2* mutant with plasmid pMK201 (Δ*pstA1*) [3]. Transformants were grown for 24 hr in 7H9 broth prior to selection of recombinants on 7H10 agar containing kanamycin (15 μg/ml) and hygromycin (50 μg/ml). Kan^R^/Hyg^R^ colonies were picked and grown in 7H9 broth without antibiotics to mid-exponential phase. Integration of the constructs at the correct chromosomal locus was confirmed by PCR on heat-inactivated cell lysates. Integration by the upstream and downstream homology regions was confirmed with the following primer pairs (S4 Table): Δ*pstA2* upstream ALN52/ALN53; Δ*pstA2* downstream ALN61/ALN62; Δ*pstS1* upstream S2KOF2/S1del2R; Δ*pstS1* downstream S1F4/S1R3; Δ*pstS3* upstream S3F3/S3R4; Δ*pstS3* downstream S3del2F/S3del2R; Δ*pstA1* upstream A1F3/A1R4; Δ*pstA1* downstream A1F4/A1R3 [3]. Clones that contained the allelic exchange vector integrated at the correct locus were plated on 7H10 agar containing 2% sucrose for counter-selection of the vector. Sucrose resistant clones were grown in 7H9 broth and isolates in which the deletion replaced the wild-type allele were identified by PCR with the following primer pairs (S4 Table): Δ*pstA2* ALN52/ALN62; Δ*pstS1* S2KOF2/S1R3; Δ*pstS3* S3F3/S3del2R; Δ*pstA1* A1F3/A1R3 [3]. The Δ*pstA2* and Δ*pstS1* mutants were complemented by electroporation with pAT208 (pMV*pstA2*) or pAT243 (pMV*pstS1*), respectively, and selection of transformants on 7H10 agar containing kanamycin (15 μg/ml). The presence of the complementing plasmid was confirmed with primers MVinsF/ALN08 (pMV*pstA2*) or pMV_3821/ProBR (pMV*pstS1*) (S4 Table).

### Southern Hybridization

Southern blotting to detect the Δ*pstA1* mutation in the Δ*pstA1*/Δ*pstA2* double mutant was done as described [3], except that genomic DNA was digested only with the restriction enzyme PstI. Southern blotting to detect the Δ*pstA2* mutation was done as described [3] except that genomic DNA from wild-type Erdman or the Δ*pstA2* mutant was digested with the restriction enzyme SacI or XhoI and a probe approximately 2 kb downstream of the Δ*pstA2* deletion was generated by PCR amplification from WT genomic DNA using primers delA2SF and delA2SR (S4 Table).

### Analysis of Phthiocerol Dimycocerosate (PDIM) Production

Analysis of PDIM production was done as described [3] by performing thin layer chromatography on extracted ^14^C propionate-labelled apolar cell wall lipids.

### Transcriptional Profiling

Bacteria grown at 37°C in 7H9 broth were maintained in early exponential phase for 4 days by daily dilution. Bacteria were harvested at OD_600_ = 0.2 and RNA was extracted and converted to labeled cDNA as described [3, 26]. Oligonucleotides (Operon) were printed in microarrays at the University of Colorado Denver microarray core. Labeled cDNAs were hybridized to the arrays overnight at 54°C as described [3]. Arrays were scanned with a GenePix400b (Molecular Devices) and spot intensities were obtained using GenePix Pro 6.0 (Molecular Devices). Data were analyzed with ArrayStar software using the Robust Multichip Averaging (RMA) with Quantile parameter for normalization. A moderated *t-*test was performed using a Benjamini Hochberg multiple testing correction and a *P* < 0.05 (5% false discovery rate) cut-off. Complete gene expression data for Δ*pstA1*, Δ*pstA2*, and Δ*pstA1*Δ*pstA2* vs. WT are reported in S1 Table. Raw gene expression data are available on the Gene Expression Omnibus (GEO) database under accession numbers GSE83812 (Δ*pstA2* and Δ*pstA1*Δ*pstA2*) and GSE36998 (Δ*pstA1*) [3].

### Quantitative RT-PCR

For confirmation of the transcriptional profiling results, bacteria were grown to mid-exponential phase (OD_600_ 0.5) in 7H9 broth and RNA was extracted as described [3]. Equivalent amounts of total RNA were treated with Turbo DNase (Ambion) and reverse transcribed to cDNA with the Transcriptor First Strand cDNA Synthesis Kit (Roche) using random hexamers for priming and the following cycling conditions: 10 min 25°C annealing, 60 min 50°C extension, 5 min 85°C heat inactivation. cDNA was stored at -20°C until real-time PCR reactions were performed. Quantitative Real Time PCR reactions were prepared with 2× FastStart Sybr Green Master Mix (Roche), 2 μl cDNA, and 0.3 μM primers and were run in absolute quantification mode on a LightCycler 480 (Roche). PCR cycling conditions were: 95°C 10 min; 45 cycles of 95°C 10 sec, 60°C 20 sec, 72°C 20 sec with data collection once per cycle during the extension phase; one cycle 95°C 5 sec, 60°C 1 min, 97°C 15 sec with a ramp rate of 0.11°C/sec to generate melting curves for confirmation of product specificity. Mock reactions (no RT) were performed on each sample to confirm the absence of genomic DNA contamination. Cp values were converted to copy numbers using standard curves for each gene. Target cDNA was internally normalized to *sigA* cDNA. Primers for real-time quantitative reverse transcriptase (RT) PCR used in this study (S5 Table) were designed using the Roche online Universal ProbeLibrary assay design center tool and were tested in standard PCR reactions using 100 *M*. *tuberculosis* genome equivalents as template.

### Mouse Infections

Male and female C57BL/6, IFN-γ^-/-^ and NOS2^-/-^ mice 6–8 weeks of age were purchased from Charles River Labs, France or Jackson Laboratory, USA. Irgm1^-/-^ mice [27] were bred under specific pathogen-free conditions at the EPFL Center of Phenogenomics. Mice were infected via the aerosol route with ~100 CFU using a custom-built aerosol chamber, as described [6]. Groups of animals (n = 4) were humanely euthanized at pre-determined time points by CO_2_ overdose for enumeration of viable CFU. Lung homogenates were serially diluted, plated on 7H10 agar, and colonies were counted after 3 to 4 weeks at 37°C. For survival experiments, animals were monitored daily following infection for signs of acute illness. To minimize animal suffering and distress, animals were humanely euthanized by CO_2_ overdose and scored as “died” when signs of severe clinical illness (ruffled fur, abnormal posture, immobility, weight loss, or respiratory distress) were observed. No animals died unexpectedly. The animal protocols used in this study were reviewed and approved by the Institutional Animal Care and Use Committee of The Rockefeller University and by the chief veterinarian of EPFL, the Service de la Consommation et des Affaires Vétérinaires of the Canton of Vaud, and the Swiss Office Vétérinaire Fédéral. All animal experiments were done in strict accordance with the recommendations in the Guide for the Care and Use of Laboratory Animals of the National Institutes of Health and the Swiss Law for the Protection of Animals.

### Phosphate Starvation

Bacteria were grown to mid-exponential phase (OD_600_ 0.5) in 7H9 broth, washed twice and then diluted to OD_600_ 0.05 in P_i_-free 7H9 broth, and incubated at 37°C. CFU were enumerated at 0, 3, 5, 7, and 10 days by plating serially diluted culture aliquots on 7H10 agar.

### Cell Wall and ROS Stress

Bacteria were grown to mid-exponential phase (OD_600_ 0.5) in 7H9 broth, diluted to OD_600_ 0.05 in fresh 7H9 broth, and incubated at 37°C after addition of 0.5% SDS or 5 mM H_2_O_2_. CFU were enumerated at 0 and 24 hr by plating serially diluted culture aliquots on 7H10 agar.

### Acid Stress

Bacteria were grown to mid-exponential phase (OD_600_ 0.5) in 7H9 broth, pelleted by centrifugation (3000 x*g*, 10 min) and resuspended in an equivalent volume of 7H9 broth at pH 4.5. Cultures were diluted to OD_600_ 0.05 in 7H9 pH 4.5 and incubated at 37°C with shaking. CFU were enumerated at 0, 3, 7, and 10 days by plating serially diluted culture aliquots on 7H10 agar.

### Western Blotting

Bacteria were grown to mid-exponential phase (OD_600_ 0.5) in 7H9 broth and collected by centrifugation (4700 x*g*, 15 min, 4°C). Cell pellets were washed twice in ice-cold PBS and resuspended in 1 ml PBS containing Complete EDTA-free Protease Inhibitors (Roche). Cells were lysed by bead beating with 0.1 mm zirconia beads (BioSpec Products). Beads and unlysed bacteria were removed by centrifugation (600 x*g*, 5 min, 4°C). Large cell debris was removed from the lysate by centrifugation (3000 x*g*, 10 min, 4°C). Lysates were passed through a Nanosep MF column with 0.2μm filter (Pall Life Sciences) by centrifugation (14,000 x*g*, 3 min, 4°C) to remove any remaining intact bacteria. Total protein concentration was quantified using the Pierce BCA Protein Concentration Assay kit (Thermo Scientific).

Equivalent amounts of whole cell lysate (4μg) were separated by sodium dodecyl sulfate polyacrylamide gel electrophoresis (SDS-PAGE) on Mini-PROTEAN TGX Any kD gels (Bio-Rad). Proteins were transferred to nitrocellulose membranes (Whatman) by electrophoresis and blocked overnight in PBS containing 0.05% Tween-20 (PBS-T) and 5% non-fat milk powder. Membranes were washed in PBS-T and probed with primary antisera in PBS-T containing 2.5% non-fat milk powder at room temperature for 1 hour. Primary antisera were used at the following dilutions: mouse α-PstS1 1:1000, mouse α-GroEL2 1:1000. Membranes were washed in PBS-T and probed with the secondary antiserum rabbit-anti-mouse conjugated to HRP (Sigma) for 1 hour at room temperature in PBS-T containing 2.5% non-fat milk powder. Membranes were washed in PBS-T and reactive bands were detected using SuperSignal West Pico substrate (Thermo Scientific). Blots were exposed to film (Blue lite autorad, GeneMate) and developed using an automated film processor.

### Statistical Analysis

Student’s unpaired *t*-test (two-tailed) was used for pairwise comparisons between WT and mutant strains of *M*. *tuberculosis*. The Mantel-Cox log-rank test was used for comparison of Kaplan-Meier plots of mouse survival. *P* values were calculated using GraphPad Prism 5.0 software (GraphPad Software, Inc.). *P* values < 0.05 were considered significant.

## Supporting Information

S1 Table Complete Gene Expression Data for Δ*pstA1*, Δ*pstA2*, and Δ*pstA1*Δ*pstA2* Mutants vs. WT and Δ*pstA1* vs. Δ*pstA1*Δ*pstA2*. (XLSX)

S2 Table Genes Differentially Expressed in the Δ*pstA2* Mutant. Gene expression ratios of Δ*pstA2* vs. WT *M*. *tuberculosis* were determined by transcriptional profiling. Genes that were dysregulated (> 2-fold change in expression) in four biological replicate experiments are listed. Ratio values > 1 indicate increased expression in Δ*pstA2* bacteria; ratio values < 1 indicate reduced expression in Δ*pstA2* bacteria.
(DOCX)

S3 Table Genes Differentially Expressed by the Δ*pstA1*Δ*pstA2* Double Mutant. Gene expression ratios of *M*. *tuberculosis* Δ*pstA1*Δ*pstA2* vs. wild-type (WT) and Δ*pstA1* vs. WT were determined by transcriptional profiling. Genes that exhibited significant dysregulation (> 2-fold, *P* < 0.05) in the Δ*pstA1*Δ*pstA2* mutant vs. WT determined from six biological replicates are listed. Ratio values > 1 indicate increased expression in the mutant; ratio values < 1 indicate reduced expression in the mutant. Genes that did not achieve either the 2-fold change in expression or *P* < 0.05 cut-offs in the Δ*pstA1* vs. WT transcriptional profiling are highlighted in light gray.
(XLSX)

S4 Table Oligonucleotide primers used for cloning and strain construction. Restriction enzyme sites are underlined. Start and stop codons for in-frame deletions are indicated in bold.
(DOCX)

S5 Table Oligonucleotide primers used for quantitative Real Time RT-PCR. (DOCX)
