# Supplementary material for: Mycobacterium tuberculosis Phosphate Uptake System Component PstA2 Is Not Required for Gene Regulation or Virulence
Source: PLoS One. 2016 Aug 24;11(8):e0161467. doi: 10.1371/journal.pone.0161467 (PMC4996455; doi:10.1371/journal.pone.0161467)
Supplement: S2 Table — Gene expression ratios of ΔpstA2 vs. WT M. tuberculosis were determined by transcriptional profiling. Genes that were dysregulated (> 2-fold change in expression) in four biological replicate experiments are listed. Ratio values > 1 indicate increased expression in ΔpstA2 bacteria; ratio values < 1 indicate reduced expression in ΔpstA2 bacteria. (DOCX) [file pone.0161467.s002.docx]

**S2 Table. Genes Potentially Differentially Expressed in the ∆*pstA2* Mutant.**

| **Rv number** | **Name** | **Description** | **Ratio (∆*pstA2*/WT)^a^** | ***P*-value^b^** |
| --- | --- | --- | --- | --- |
| Rv0108c | *rv0108c* | Hypothetical protein | 2.1 | 1.0 |
| Rv0119 | *fadD7* | Fatty acid-CoA ligase | 2.3 | 1.0 |
| Rv0160c | PE4 | PE-family protein | 0.4 | 1.0 |
| Rv0194 | *rv0194* | Probable multidrug efflux pump | 2.2 | 1.0 |
| Rv0195 | *rv0195* | Transcriptional regulator (LuxR/UhpA family) | 0.5 | 1.0 |
| Rv0261c | *narK3* | Nitrite extrusion protein | 0.5 | 1.0 |
| Rv0280 | PPE3 | PPE-family protein | 2.2 | 1.0 |
| Rv0307c | *rv0307c* | Hypothetical protein | 0.5 | 1.0 |
| Rv0312 | *rv0312* | Conserved hypothetical protein | 2.8 | 1.0 |
| Rv0336 | *rv0336* | Conserved hypothetical protein | 2.1 | 1.0 |
| Rv0350 | *dnaK* | 70 kD heat shock protein chaperone | 0.4 | 1.0 |
| Rv0440 | *groEL2* | 60 kD chaperonin 2 | 0.3 | 1.0 |
| Rv0689c | *rv0689c* | Conserved hypothetical protein | 0.3 | 1.0 |
| Rv0696 | *rv0696* | Glycosyltransferase | 2.2 | 1.0 |
| Rv0767c | Rv0767c | Conserved hypothetical protein | 0.5 | 1.0 |
| Rv0814c | *sseC2* | Thiosulfate sulfurtransferase | 2.2 | 1.0 |
| Rv0936 | *pstA2* | Phosphate ABC transporter | 0.1 | 0.04 |
| Rv0956 | *purN* | Phosphoribosylglycinamide formyltransferase | 2.1 | 1.0 |
| Rv1000c | *rv1000c* | Conserved hypothetical protein | 0.5 | 1.0 |
| Rv1029 | *kdpA* | Potassium-transporter ATPase | 0.5 | 1.0 |
| Rv1045 | *rv1045* | Hypothetical protein | 0.4 | 1.0 |
| Rv1120c | *rv1120c* | Conserved hypothetical protein | 0.4 | 1.0 |
| Rv1209 | *rv1209* | Conserved hypothetical protein | 0.4 | 1.0 |
| Rv1225c | *rv1225c* | Conserved hypothetical protein | 0.3 | 1.0 |
| Rv1226c | *rv1226c* | Possible membrane protein | 0.3 | 1.0 |
| Rv1388 | *mihF* | Putative integration host factor | 0.3 | 1.0 |
| Rv1505c | *rv1505c* | Conserved hypothetical protein | 0.5 | 1.0 |
| Rv1544 | *rv1544* | Probable ketoacyl reductase | 0.5 | 1.0 |
| Rv1641 | *infC* | Initiation factor IF-3 | 0.5 | 1.0 |
| Rv1983 | PE_PGRS35 | PE_PGRS-family protein | 0.5 | 1.0 |
| Rv2013 | *rv2013* | Transposase | 0.5 | 1.0 |
| Rv2123 | PPE37 | PPE family protein | 2.3 | 1.0 |
| Rv2251 | *rv2251* | Possible flavoprotein | 0.4 | 1.0 |
| Rv2270 | *lppN* | Possible lipoprotein | 0.5 | 1.0 |
| Rv2275 | *rv2275* | Conserved hypothetical protein | 0.5 | 1.0 |
| Rv2276 | *cyp121* | Probable cytochrome P-450 | 0.4 | 1.0 |
| Rv2312 | *rv2312* | Hypothetical protein | 0.4 | 1.0 |
| Rv2316 | *uspA* | Sugar transport protein | 0.4 | 1.0 |
| Rv2351c | *plcA* | Phospholipase C precursor | 2.1 | 1.0 |
| Rv2369c | *rv2369c* | Hypothetical protein | 2.1 | 1.0 |
| Rv2423 | *rv2423* | Hypothetical protein | 3.7 | 1.0 |
| Rv2425c | *rv2425c* | Conserved hypothetical protein | 3.6 | 1.0 |
| Rv2477c | *rv2477c* | Probable macrolide transport ATP binding protein | 0.5 | 1.0 |
| Rv2698 | *rv2698* | Conserved hypothetical protein | 0.4 | 1.0 |
| Rv2772c | *rv2772c* | Conserved hypothetical protein | 0.5 | 1.0 |
| Rv2787 | *rv2787* | Conserved hypothetical protein | 3.2 | 1.0 |
| Rv2874 | *dipZ* | C-type cytochrome biogenesis | 2.0 | 1.0 |
| Rv2926c | *rv2926c* | Conserved hypothetical protein | 0.5 | 1.0 |
| Rv3167c | *rv3167c* | Putative transcriptional regulator | 0.4 | 1.0 |
| Rv3170 | *aohF* | Probable flavin-containing monoamine oxidase | 2.4 | 1.0 |
| Rv3284 | *rv3284* | Conserved hypothetical protein | 0.5 | 1.0 |
| Rv3383c | *idsB* | Possible polyprenyl synthetase | 2.2 | 1.0 |
| Rv3384c | *vapC46* | Possible VapC toxin | 2.9 | 1.0 |
| Rv3513c | *fadD18* | Fatty acid-CoA ligase | 0.5 | 1.0 |
| Rv3706c | *rv3706c* | Hypothetical protein | 2.2 | 1.0 |
| Rv3746c | PE34 | PE-family protein | 2.0 | 1.0 |
| Rv3900c | *rv3900c* | Conserved hypothetical protein | 0.5 | 1.0 |
| MT0291.4 |  | Hypothetical protein | 0.5 | 1.0 |
| MT0740.1 |  | Hypothetical protein | 0.3 | 1.0 |
| MT0910.4 |  | Hypothetical protein | 0.3 | 1.0 |
| MT1264.1 |  | Hypothetical protein | 2.0 | 1.0 |
| MT1432 |  | Hypothetical protein | 0.4 | 1.0 |
| MT2375 |  | Hypothetical protein | 2.2 | 1.0 |
| MT2396 |  | Hypothetical protein | 2.4 | 1.0 |
| MT2405 |  | Hypothetical protein | 0.5 | 1.0 |
| MT2514 |  | Hypothetical protein | 4.0 | 1.0 |
| MT2518.1 |  | Hypothetical protein | 0.4 | 1.0 |
| MT3174.1 |  | Hypothetical protein | 0.5 | 1.0 |
| MT3290.1 |  | Conserved hypothetical protein | 3.1 | 1.0 |
| MT3615.4 |  | Hypothetical protein | 0.4 | 1.0 |

**^a^** Gene expression ratios of ∆*pstA2* vs. WT *M. tuberculosis* were determined by transcriptional profiling. Genes that were dysregulated (> 2-fold) in four biological replicate experiments are listed. Ratio values > 1 indicate potentially increased expression in ∆*pstA2* bacteria; ratio values < 1 indicate potentially reduced expression in ∆*pstA2* bacteria.

**^b^** *P*-value determined with a moderated *t*-test using a Benjamini Hochberg multiple testing correction.
