# Supplementary material for: Mycobacterium tuberculosis Phosphate Uptake System Component PstA2 Is Not Required for Gene Regulation or Virulence
Source: PLoS One. 2016 Aug 24;11(8):e0161467. doi: 10.1371/journal.pone.0161467 (PMC4996455; doi:10.1371/journal.pone.0161467)
Supplement: S4 Table — Restriction enzyme sites are underlined. Start and stop codons for in-frame deletions are indicated in bold. (DOCX) [file pone.0161467.s004.docx]

**S4 Table. Oligonucleotide primers used for cloning and strain construction.** Restriction enzyme sites are underlined. Start and stop codons for in-frame deletions are indicated in bold.

| Name | Sequence 5’-3’ |
| --- | --- |
| pstA2KOF1 | CCTTAATTAACGGCGACCCGGGCAACGGGGAGGGC |
| pstA2KOR1 | GAAGGCCAAC**TCA**AACGCC**CAT**GTCAGATGCCGCGTCCCACCGGAAGC |
| pstA2KOF2 | **ATG**GGCGTT**TGA**GTTGGCCTTCGAGCGCGCCTTCACGCTGGC |
| pstA2KOR2 | GGGGCGCGCCGATGATGTTCGACCGCAGCTACTTTT |
| ALN52 | GCGATGGGTGCGATCAGGCTCAACG |
| ALN53 | CAAACTCGAAGGTGGGCAGGCATTTAC |
| ALN61 | TGCTGATGGTGATCACGTTGCTGA |
| ALN62 | TCGCGGACAATCAAGGCATACTCA |
| delA2SF | CGACAGGCTACGTTGAAAGC |
| delA2SR | GCCGACAACGCCAACTGC |
| A2P1F | GTAGCTGCAGGAGCCGCTGGAGTGGAGCT |
| A2P1R | GTACAGATCTCGCAGGGTACTACATGCTCCT |
| C1A2F | GTACAGATCTATGACCGATTCCACCAACTTCG |
| C1A2R | GTACGTCGACTCAAACGTCCCAACGCCT |
| MVinsF | AGCGAGGACAACTTGAGC |
| ALN08 | CCTAGGGCCCATGTCAGATGCCGCGT |
| S1KOF1 | TTAATTAAGCTGTTCCAGCGCCCGAATCCG |
| S1KOR1 | CCTAGGAATTTT**CAC**GCCATACCTTTCT |
| S1KOF2 | CCTAGGTCCAGC**TAG**CCTCGTTGACCAC |
| S1KOR2 | GGCGCGCCAGCGATGTGATGAGCGATGAAC |
| S2KOF2 | CCTAGGGAACTTCACGCAACTCCTCTCG |
| S1del2R | CTATCCCACCCAACCACC |
| S1F4 | TACGTCGCCGGACTGTCG |
| S1R3 | GCCGCGCAAGTAGTCAGC |
| S3KOF1 | TTAATTAAAAGCTCCGACGGCCTGACCAG |
| S3KOR1 | CCTAGGGAGTTT**CAA**TTCAGTTCCTAAC |
| S3KOF2 | CCTAGGATCGCC**TGA**TCTGAGGTTGACG |
| S3KOR2 | GGCGCGCCCCCAAGTTGCGATTGAGAAA |
| S3F3 | AGGTGCTTAGCAGCGTGTTG |
| S3R4 | CGCGGCCAGCTTGAACAG |
| S3del2F | GCCGAAACGGTAACAAGC |
| S3del2R | ATCATCACCGACAGCACG |
| ProBF | AGCTCTAGAGAGCCGCTGGAGTGGAGCT |
| ProBR | AGCGAATTC**CAT**CGGCGCAGGGTACTACAT |
| S1CF2 | AGCGAATTCAAGCGCGGAAAT**TGA**AGAGCAC |
| S1CR | AGCAAGCTTCCCGACGGAGGTTGCTGTC |
| pMV_3821 | CCATTTCCGCTGAATATCGTG |
