# Supplementary material for: Mycobacterium tuberculosis Phosphate Uptake System Component PstA2 Is Not Required for Gene Regulation or Virulence
Source: PLoS One. 2016 Aug 24;11(8):e0161467. doi: 10.1371/journal.pone.0161467 (PMC4996455; doi:10.1371/journal.pone.0161467)
Supplement: S5 Table — (DOCX) [file pone.0161467.s005.docx]

**S5 Table. Oligonucleotide primers used for quantitative Real Time RT-PCR.**

| **Gene** | **Forward primer sequence (5’-3’)** | **Reverse primer sequence (5’-3’)** |
| --- | --- | --- |
| *sigA* | CGCGACATGATGTGGATCT | GGCTAGCGAAACCACCAG |
| *pstA2* | TGTGCTGGTGCAGGACTC | AAATCCCGGTCAACACACTC |
| *pe4* | ACCCCGAGTACGTGACAGAC | ACAGGCCTTGCGAGACAG |
| *narK3* | CTGTTGATTCCGACCGTTG | ACCGCACACCAGATACGG |
| *rv0307* | CCTGCTGACCTACGCTGTC | CCACGTATTCGGCAAGGTA |
| *rv1045* | GCTTCTTGAAGGACTGTTGCTC | CGCACCTGCATAGATCAGC |
| *rv1505* | CAACACTATCCAGCCATTCGT | ATATGATTGCCGCTCCACA |
| *udgA* | TCAACCCCGACCGTATCGT | CTCGCGGACGGCTACCT |
| *mgtA* | GCGGTCTACCAAACCGATGT | CGTGCTGTCATCGGAATGC |
| *rv0784* | CGATGTCGACGCGTTCTG | GAGCCACCAGCAACGATACC |
| *cpsY* | AACCGAACCGATGTACGC | GGCGACCAAAACAGGAGA |
| *lipM* | CGACTCATCCACGTCATCC | GGCCTATATGCGTTGATCGT |
| *rv3371* | CGATCTTGATAAAAATCCACCAT | CATTGTTAGCGAAGGCACTG |
| *rv0566* | CGGACTCATCGTTCGACAT | CCTGGTTGAGTGCGTTGTC |
| *icl1* | GCAGGAATGGGACACGAA | GTCCTCGGCGGAGTAGGT |
| *esxD* | CCAGGAAAACGTCATGAACC | ACCTTGTTCAATTCATTGGTGA |
